# Muscle Damage Indicated by Maximal Voluntary Contraction Strength Changes From Immediately to 1 Day After Eccentric Exercise of the Knee Extensors

**DOI:** 10.3389/fphys.2021.775157

**Published:** 2021-11-17

**Authors:** Mikio Shoji, Ryoichi Ema, Kazunori Nosaka, Akihiro Kanda, Kosuke Hirata, Ryota Akagi

**Affiliations:** ^1^Graduate School of Engineering and Science, Shibaura Institute of Technology, Saitama, Japan; ^2^Faculty of Sport Science, Shizuoka Sangyo University, Iwata-shi, Japan; ^3^School of Medical and Health Sciences, Edith Cowan University, Joondalup, WA, Australia; ^4^Mizuno Corporation, Osaka, Japan; ^5^Faculty of Sport Sciences, Waseda University, Tokorozawa-shi, Japan; ^6^College of Systems Engineering and Science, Shibaura Institute of Technology, Saitama, Japan

**Keywords:** maximal voluntary isometric contraction, recovery rate, muscle soreness, voluntary activation, potentiated doublet torque, shear modulus

## Abstract

The present study examined if the magnitude of changes in indirect muscle damage markers could be predicted by maximal voluntary isometric contraction (MVIC) torque changes from immediately to 1 day after eccentric exercise. Twenty-eight young men performed 100 maximal isokinetic (60°/s) eccentric contractions of the knee extensors. MVIC torque, potentiated doublet torque, voluntary activation (VA) during MVIC, shear modulus of rectus femoris (RF), vastus medialis and lateralis, and muscle soreness of these muscles were measured before, immediately after, and 1–3 days post-exercise. Based on the recovery rate of the MVIC torque from immediately to 1-day post-exercise, the participants were placed to a recovery group that showed an increase in the MVIC torque (11.3–79.9%, *n* = 15) or a no-recovery group that showed no recovery (−71.9 to 0%, *n* = 13). No significant difference in MVIC torque decrease immediately post-exercise was found between the recovery (−33 ± 12%) and no-recovery (−32 ± 9%) groups. At 1–3 days, changes in MVIC torque (−40 to −26% vs. −22 to −12%), potentiated doublet torque (−37 to −22% vs. −20 to −9%), and proximal RF shear modulus (29–34% vs. 8–15%) were greater (*p* < 0.05) for the no-recovery than recovery group. No significant group differences were found for muscle soreness. The recovery rate of MVIC torque was correlated (*p* < 0.05) with the change in MVIC torque from baseline to 2 (*r* = 0.624) or 3 days post-exercise (*r* = 0.526), or peak change in potentiated doublet torque at 1–3 days post-exercise from baseline (*r* = 0.691), but not correlated with the changes in other dependent variables. These results suggest that the recovery rate of MVIC torque predicts changes in neuromuscular function but not muscle soreness and stiffness following eccentric exercise of the knee extensors.

## Introduction

Unaccustomed eccentric exercises induce muscle damage that is accompanied by symptoms such as prolonged strength loss, delayed onset muscle soreness (DOMS), and increased muscle stiffness (Clarkson et al., 1992). However, the time-course changes in these symptoms following eccentric exercise are different from each other. For example, the decrease in maximal voluntary isometric contraction (MVIC) torque is the greatest immediately after (Baroni et al., 2010) or 1 day after exercise (Crameri et al., 2007), whereas muscle soreness peaks at 1–3 days post-exercise (Chapman et al., 2006). Muscle strength is considered the best indirect muscle damage marker (Paulsen et al., 2012), but a decrease in muscle strength is not solely due to muscle damage, since it is also a consequence of neuromuscular fatigue (Souron et al., 2018). In addition, changes in muscle strength are affected by central and peripheral functions, with prolonged strength loss being mainly due to peripheral damage (Hubal et al., 2007; Souron et al., 2018). It is important to predict the magnitude of symptoms of muscle damage such as DOMS and loss of muscle function with the assessment of central and peripheral factors in days after eccentric exercises.

Chen et al. (2020) compared changes in several indirect markers of muscle damage following three different intensities of eccentric exercise of the elbow flexors (10, 50, and 100% of MVIC torque) that were matched for the magnitude of decrease in MVIC torque at 1-day post-exercise. They reported that the recovery rate of MVIC torque from immediately to 1-day post-exercise was highly correlated with not only the decrease in MVIC torque at 2–5 days post-exercise but also the peak changes in other muscle damage markers such as range of motion, muscle soreness, circumference, and plasma creatine kinase activity (Chen et al., 2020). This suggests that the recovery rate of MVIC torque from immediately to 1-day post-exercise predicts the magnitude of muscle damage symptoms and its recovery time course for the elbow flexors. However, it is not known whether this is also the case for other muscles.

Some studies reported that the MVIC torque did not recover or further decreased from immediately to 1–2 days after maximal voluntary eccentric exercise of the knee extensors (McHugh and Pasiakos, 2004; Crameri et al., 2007; Paschalis et al., 2011). For example, the decrease in MVIC torque was the greatest at 1 day after 120 (McHugh and Pasiakos, 2004) or 210 (Crameri et al., 2007) maximal knee extensor eccentric contractions. Paschalis et al. (2011) reported that the MVIC torque reduction after 75 maximal knee extensor eccentric contractions was similar between immediately and 2 days post-exercise. Thus, the recovery rate of MVIC torque from immediately to 1-day post-exercise may not be valid to predict the time course of muscle damage recovery for knee extensor eccentric exercises. However, this has not been systematically investigated in the previous studies. Chen et al. (2020) speculated that the largest recovery rate of MVIC torque from immediately to 1-day post-exercise in the group of the lowest intensity (10% of MVIC torque) was due more to neuromuscular fatigue than damage to muscle fibers and the extracellular matrix. However, no neuromuscular function assessments were included in the study. It is interesting to compare the time-course changes in neuromuscular parameters between individuals who show a recovery and who do not show a recovery of MVIC torque from immediately to 1-day post-exercise.

Therefore, the purposes of the present study were (1) to compare individuals who showed recovery and no-recovery of MVIC torque from immediately to 1 day after knee extensor eccentric exercise for their indirect muscle damage characteristics, and (2) to examine the relationships between the recovery rate of MVIC torque and changes in several indirect muscle damage markers. It was hypothesized that (1) compared to the recovery group, the no-recovery group would demonstrate greater decreases in voluntary activation (VA) and evoked torque, and greater increases in muscle soreness and stiffness at 1–3 days post-exercise, and (2) the recovery rate of MVIC torque from immediately to 1-day post-exercise would be significantly correlated with the magnitude of changes in the other indirect muscle damage markers such as DOMS and increased muscle stiffness.

## Materials and Methods

### Participants

*A priori* power analysis was performed to calculate the sample size necessary for detecting possible differences in the changes in indirect muscle damage markers between two groups (outlined below) by a two-way mixed design analysis of variance (ANOVA) using the G^∗^Power statistical power analysis (G^∗^Power 3.1.9.6, Kiel University, Germany). Based on the study by Chen et al. (2020), we estimated the effect size to be 0.25 for the difference in MVIC torque between groups. With α of 0.05, power (1 − β) of 0.80, a correlation among repeated measures of 0.5, and a non-sphericity correction of 1, the total sample size was estimated to be 24. Considering the estimation error, 28 healthy young men who had not performed regular resistance training of lower limb muscles and had not experienced muscle soreness of knee extensors in the past 6 months were recruited from students in a university. They had no history of previous muscle, joint, or bone injuries of the lower limb. During the experimental period, the participants were requested not to take any nutritional supplements. The mean ± standard deviation (SD) of age, height, and body mass was 22.3 ± 1.1 years, 171.7 ± 6.3 cm, and 63.9 ± 7.9 kg, respectively. This study was approved by the Ethics Committee of Shibaura Institute of Technology and was performed according to the Declaration of Helsinki. Participants were informed of this study’s purpose and potential risks and provided written informed consent. No familiarization session was included in the present study because only a small number of MVIC contractions could affect the magnitude of muscle damage (Chen et al., 2013).

### Eccentric Exercise

All participants performed maximal eccentric exercise of the knee extensors using their right legs, regardless of leg dominance, because the magnitude of muscle damage is not affected by leg dominance (Hody et al., 2013). Each participant lay in a supine position on a bed of an isokinetic dynamometer (CON-TREX MJ, PHYSIOMED, Germany), and the trunk was secured to it by straps. This exercise position was selected because of greater damage indicated by decreases in muscle strength and increases in shear modulus of the knee extensor rectus femoris (RF) was observed after eccentric exercise in a supine compared with a sitting position in our previous study (Ema et al., 2021). The exercise consisted of 10 sets × 10 maximal voluntary eccentric contractions and was performed at an angular velocity of 60°/s from an extended knee position at 40° (anatomical position = 0°) to a flexed knee joint position at 110° (i.e., range of motion of the knee joint during the exercise was 70°) on the isokinetic dynamometer. After each contraction, the knee joint was passively returned to the extended position at a velocity of 20°/s, which provided a 3.5-s rest between contractions. A 2-min rest was provided between sets. The participants were verbally encouraged to maximally resist the movements of the isokinetic dynamometer to flex the knee joint. Torque signals were recorded at 2 kHz using an A/D converter (PowerLab16/35; ADInstrument, Australia) and stored on a personal computer. The exercise volume was determined as the area under the torque-time curve during the eccentric exercise.

### Dependent Variables

The dependent variables included MVIC torque of the knee extensors, potentiated doublet torque, VA during knee extensor MVIC, shear modulus of the rectus femoris in the proximal and distal regions (RFp and RFd), vastus medialis (VM) and vastus lateralis (VL), and muscle soreness of these muscles by a visual analog scale (VAS) (Doguet et al., 2016; Ema et al., 2021). These measurements were performed before, immediately after (MVIC torque, potentiated double torque, and VA), and 1–3 days after eccentric exercise.

### Maximal Voluntary Isometric Contraction Torque, Potentiated Doublet Torque, and Voluntary Activation

The MVIC torque of the knee extensors was measured in a supine position with the knee flexed at 90° on the isokinetic dynamometer. After several warm-up contractions, the participants performed two knee extensor MVICs for 4 sec each with a 1-min rest between contractions. If more than 10% difference in the two MVIC torque values was found, one more MVIC measure was performed for all time points except immediately post-exercise. Immediately post-exercise, the measurement of MVIC torque was taken once, because other measurements were required to be taken in a short period of time. Participants were verbally encouraged during the measurements. Torque signals were recorded at 2 kHz using an A/D converter (PowerLab16/35; ADInstrument, Australia) and stored on a personal computer. The mean coefficient of variation (CV) of two or three corresponding trials for each participant at baseline was 2.8%. The highest value was used for further analyses. The recovery rate of MVIC torque from immediately to 1 day after the exercise was calculated using the following formula: recovery rate = ([MVIC torque at 1 day − MVIC torque at immediately post-exercise]/[MVIC torque at baseline − MVIC torque at immediately post-exercise]) × 100 (Chen et al., 2020).

In the same trials as the MVIC torque measurements, VA during knee extensor MVIC and potentiated doublet torque were assessed using a twitch interpolation technique (Allen et al., 1995). The femoral nerve was stimulated using a constant current variable voltage stimulator (DS7AH, Digitimer Ltd., United Kingdom) with a controller (SEN-3401, Nihon Kohden, Japan). To stimulate percutaneously the femoral nerve, a cathode (2 cm × 2 cm) was placed at the femoral triangle and an anode (4 cm × 5 cm) was placed at the midway between the superior aspect of the greater trochanter and the inferior border of the iliac crest. The supramaximal stimulus intensity was determined by increasing the stimulation current in 10-mA steps, starting from 30 mA until plateaus in the twitch torque occurred, and the intensity was set at 120% of the current (range, 60–264 mA) with the voltage set at a maximum of 400 V. After the intensity was set, supramaximal doublet stimulations (duration: 200 μs, interval: 10 ms) were provided during and 2 sec after the knee extensor MVIC. The VA was estimated using the following formula: VA = (1 − [superimposed doublet torque/potentiated resting doublet torque]) × 100. The mean CV of two corresponding trials for potentiated doublet torque and VA at baseline were 1.3 and 3.6%, respectively. VA and potentiated doublet torque observed in the selected trial for evaluating MVIC torque was used for further analyses.

### Muscle Soreness

The extent of muscle soreness of RFp, RFd, VM, and VL were quantified using a VAS that had a 100-mm continuous line with “no pain” on one end (0 mm) and “unbearable pain” on the other end (100 mm). The measurement sites of muscle soreness of RFp, RFd, VM, and VL were 30, 50, 70, and 50% proximal of the thigh-length from the greater trochanter to the popliteal crease, respectively. During the measurement, the participants sat on the dynamometer with the hip joint at 40° and the knee joint at 90° flexion and relaxed their knee extensors. The participants were asked to rate their perceived soreness on the VAS when investigator palpated these muscles by fingers.

### Muscle Shear Modulus

According to a previous study (Ema et al., 2021), resting muscle shear modulus of RFp, RFd, VM, and VL were calculated from the shear wave speed obtained using an elastography mode of the ultrasound apparatus (ACUSON S2000, Siemens Medical Solutions, Germany) with a linear array transducer (9L4 Transducer, 4–9 MHz, Siemens Medical Solutions, United States). Through the measurements, frequency (7 MHz), gain (0 dB), and depth (8 cm) were unchanged. During the measurement, the participants sat on the dynamometer in the same posture of muscle soreness. Imaging positions for those muscles were the same as those of muscle soreness. The ultrasound probe orientation was aligned to the fascicle direction of each muscle. To facilitate consistency between measurements, these probe locations were marked on the skin and referred to throughout the experiment. In the measurement after the eccentric exercise, the elastographic images were captured while referring to the marks and B-mode images taken at the baseline in order to acquire them at the same locations within the muscles.

The elastographic images were analyzed in the same way as that described in a previous study (Hirata et al., 2020). Briefly, a region of interest (ROI) on the muscle was made as large as possible within the color-coded area of the elastographic image while excluding non-target tissue (e.g., subcutaneous adipose tissues, aponeuroses, non-target muscles, etc.) using image processing software (Image J, NIH, United States). The average value of shear modulus over the ROI was calculated for each image using our original analysis software written in MATLAB (MATLAB R2018a; Math Works, Natick, MA), which can convert the Red-Green-Blue color model values of each pixel within ROI into shear wave speed values according to the color scale of the elastographic images. The shear modulus of each pixel was calculated as the product of ρ and v^2^, where ρ is the tissue density [1.084 g/cm^3^; the mean of the two values reported in a previous study (Ward and Lieber, 2005)], and v is the shear wave speed. The mean CVs of three images for RFp, RFd, VM, and VL at baseline was 3.7, 3.1, 2.4, and 4.3%, respectively. The mean value of the three images was used for further analyses.

### Habitual Physical Activity

After the full recovery of muscle soreness (1–2 weeks after the completion of the experiment), the participants were requested to conduct their routine daily activities while wearing a device (Active style Pro HJA-750C, Omron Health Care, Japan) for 11 days. The participants were instructed to wear the device while performing the activities of their daily lives, except for time spent bathing or sleeping. If the participants did not wear the device for at least 500 min in a day, the data were excluded from the analyses. The days analyzed ranged between 4 and 11 days, which was sufficient for further analyses (Watanabe et al., 2020). Due to the influence of the COVID-19 pandemic or personal reasons, data were not obtained from some participants. The mean magnitude of physical activity was assessed at three levels: low [<3.0 metabolic equivalents (METs)], moderate (3.0–5.9 METs), and vigorous intensities (>6.0 METs) (Ema et al., 2017).

### Statistical Analyses

A k-cluster analysis was performed to separate the participants into 2 groups (recovery and no-recovery) based on the changes in MVIC torque from immediately to 1-day post-exercise (i.e., recovery rate = [MVIC torque at 1-day post-exercise − MVIC torque at immediately post-exercise]/[MVIC torque at baseline − MVIC torque at immediately post-exercise] × 100. The normality of the data was checked by the Shapiro-Wilk test, and the data of each parameter were not normally distributed for at least one of the time points. Therefore, when comparing the groups for changes in the variables over time, all data except for the VAS of muscle soreness were log-transformed before analyses. The muscle soreness data were rank-transformed before analysis.

The baseline values of the dependent variables were compared between the groups by independent *t*-test. Changes in the dependent variables over time were compared between groups by a two-way (group × time) mixed designed ANOVA. To compare the peak changes in muscle soreness and shear modulus among muscles, a two-way (group × muscle) ANOVA was performed. A two-way ANOVA (group × intensity) was conducted with physical activity. When a significant interaction effect or a main effect of time and/or muscle was found, a Bonferroni *post hoc* test was performed. For dependent variables that showed significant group differences at a time point, Cohen’s d (d) in between-subject designs (Lakens, 2013) was determined. We interpreted the group difference to be substantial when d > 0.60 (Ema et al., 2021). Using the data of all participants (*n* = 28), Pearson product-moment correlation coefficient (r) was calculated to examine the relationships between the recovery rate of MVIC torque from immediately to 1 day and the change in MVIC torque from baseline to 2–3 days post-exercise, peak changes at 1–3 days post-exercise in potentiated doublet torque, peak muscle soreness and the largest changes in the shear modulus. The significance level was set at *P* < 0.05. The data were presented as mean ± SD. All statistical analyses were performed using statistical analysis software (SPSS 24.0, IBM, United States).

## Results

### Grouping

There was a distinct difference in MVIC torque change from immediately to 1 day post-exercise among participants (Table 1). Participants were classified into the recovery group (*n* = 15, cluster center: 36.5% of recovery rate) and the no-recovery group (*n* = 13, cluster center: −25.2% of recovery rate). There were no significant differences in the baseline values of the MVIC torque and other dependent variables between the groups.

**TABLE 1:** Maximal voluntary isometric contraction (MVIC) torque at baseline, immediately (0) and 1 day after eccentric exercise of the knee extensors, and recovery rate from immediately to 1-day post-exercise ([MVIC torque at 1-day post-exercise − MVIC torque at immediately post-exercise]/[MVIC torque at baseline − MVIC torque at immediately post-exercise] × 100) for all participants (*n* = 28).

		**MVIC torque (Nm)**			**Recovery rate (%)**
**Group**	**Participants ID**	**Baseline**	**0**	**1 day**	
Recovery	1	182.6	123.9	167.6	74.4
	2	215.0	153.4	193.1	64.4
	3	170.4	79.5	109.1	32.5
	4	276.5	192.0	228.3	42.9
	5	164.3	106.8	127.3	35.7
	6	167.0	140.9	161.7	79.9
	7	147.0	116.5	134.7	59.5
	8	228.1	110.6	132.2	18.4
	9	239.1	161.7	182.9	27.4
	10	172.4	122.4	133.2	21.6
	11	352.0	190.8	212.0	13.2
	12	157.2	89.0	96.9	11.7
	13	213.8	170.2	180.2	23.0
	14	210.4	179.8	189.5	31.7
	15	189.3	115.7	124.0	11.3
No-recovery	16	187.4	114.9	114.9	0
	17	196.8	137.2	135.4	–3.0
	18	212.0	161.0	156.5	–8.8
	19	218.0	124.7	118.7	–6.4
	20	143.2	115.4	111.3	–14.7
	21	310.6	188.9	178.7	–8.5
	22	212.5	142.5	133.8	–12.4
	23	172.3	105.6	93.4	–18.3
	24	151.6	92.7	81.4	–19.2
	25	140.6	112.6	98.6	–50.0
	26	198.8	135.8	108.2	–44.0
	27	185.8	149.0	123.0	–70.7
	28	156.1	92.0	45.8	–71.9

*Based on the recovery rate, the participants were placed into two groups; Recovery and No-recovery.*

### Comparison Between Groups for Changes in Dependent Variables

#### Maximal Voluntary Isometric Contraction Torque

**FIGURE 1:**
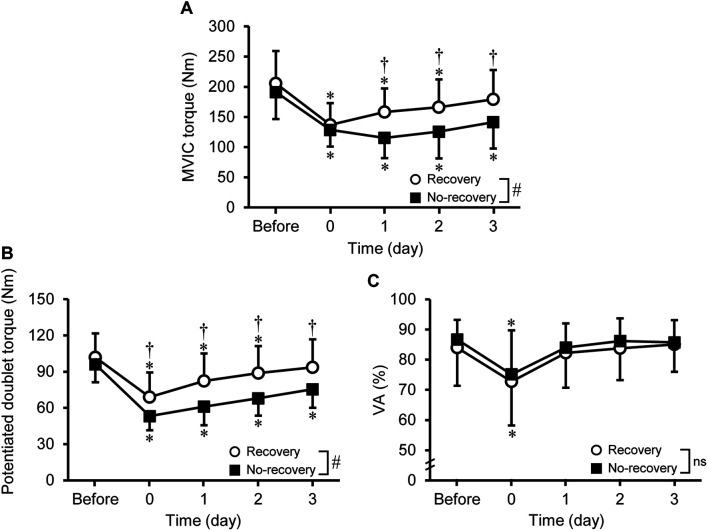
Changes (mean ± SD) in maximal voluntary isometric contraction (MVIC) torque **(A)**, potentiated doublet torque **(B)**, and voluntary activation (VA) during the MVIC **(C)** before, immediately after (0), and 1–3 days after eccentric exercise for the recovery and no-recovery groups. * Indicates a significant difference from baseline. † Shows a significant difference between groups. # Demonstrates a significant interaction effect. ns shows no significant interaction effect.

A significant group × time interaction effect was found [*F*_(2.751, 71.532)_ = 9.047, *P* < 0.001] for changes in MVIC torque over time (Figure 1A). At immediately post-exercise, MVIC torque was not significantly different between the recovery and no-recovery groups, whereas the values at 1–3 days post-exercise were lower (*P* = 0.006–0.032) for the no-recovery group (115.4–141.4 Nm) than the recovery group (158.2–179.4 Nm, *d* = 0.82–1.17). The MVIC torque recovered to the baseline at 3 days post-exercise for the recovery group (*P* = 0.071) but not for the no-recovery group (*P* < 0.001).

#### Potentiated Doublet Torque

A significant group × time interaction effect [*F*_(2.944, 76.546)_ = 6.047, *P* < 0.001] was evident for changes in potentiated doublet torque (Figure 1B). Potentiated doublet torque significantly decreased from the baseline to immediately and 1–2 days post-exercise (−20 to −9%; *P* ≤ 0.010) for the recovery group and to immediately and 1–3 days post-exercise (−37 to −22%; *P* < 0.001) for the no-recovery group. At immediately and 1–3 days post-exercise, potentiated doublet torque was greater (*P* = 0.010–0.031) for the recovery (68.9–93.6 Nm) than no-recovery group (53.1–75.4 Nm, *d* = 0.91–1.09).

#### Voluntary Activation

No significant group × time interaction effect was observed [*F*_(2.411, 62.673)_ = 0.073, *P* = 0.954], but a significant effect of time was found [*F*_(2.411, 62.673)_ = 10.849, *P* < 0.001]. The VA significantly decreased at immediately post-exercise only (−12.4 ± 18.7%).

#### Muscle Soreness

**FIGURE 2:**
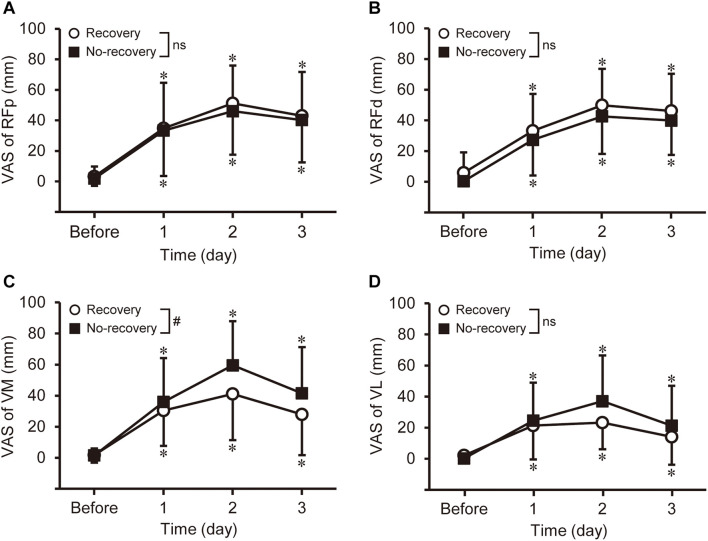
Changes in muscle soreness of the rectus femoris in the proximal and distal regions (RFp: **A** and RFd: **B**), vastus medialis (VM: **C**), and vastus lateralis (VL: **D**) assessed by a 100-mm visual analog scale (VAS) before and 1–3 days after eccentric exercise for the recovery and no-recovery groups. * Shows a significant difference from baseline. # Indicates a significant interaction effect. ns shows no significant interaction effect.

No significant group × time interaction effect was evident for muscle soreness of RFp [*F*_(3, 78)_ = 0.971, *P* = 0.411], RFd [*F*_(3, 78)_ = 0.344, *P* = 0.794], and VL [*F*_(2.580, 67.077)_ = 2.861, *P* = 0.051], but a significant time effect was found [RFp: *F*_(2.563, 66.634)_ = 67.031, *P* < 0.001; RFd: *F*_(3, 78)_ = 72.857, *P* < 0.001; VL: *F*_(3, 78)_ = 42.299, *P* < 0.001]. For VM, a significant group × time interaction effect was observed [*F*_(2.358, 61.300)_ = 3.031, *P* = 0.047], and time effect was significant for each group [*F*_(3, 24)_ = 23.428–30.918, *P* < 0.001]. As shown in Figure 2, muscle soreness developed in all muscles after exercise, but no significant differences in the changes in the VAS were found among muscles and between groups.

#### Shear Modulus

**FIGURE 3:**
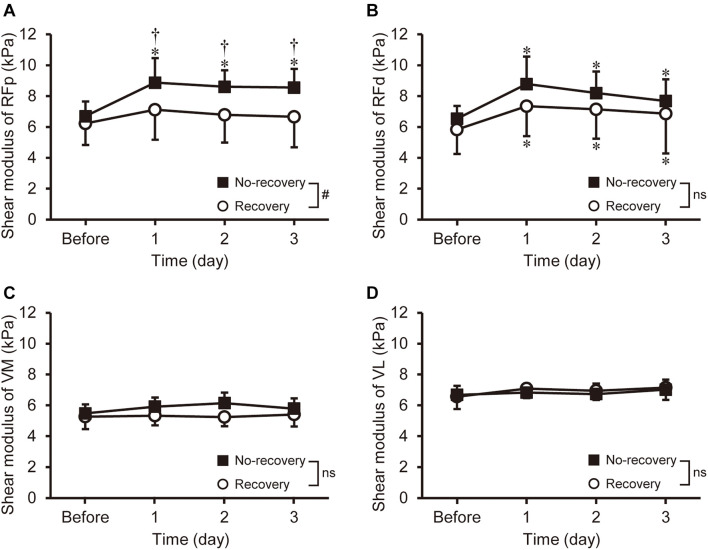
Changes (mean ± SD) in shear modulus of the rectus femoris in the proximal and distal regions (RFp: **A** and RFd: **B**), vastus medialis (VM: **C**), and vastus lateralis (VL: **D**) before and 1–3 days after eccentric exercise for the recovery and no-recovery groups. * Indicates a significant difference from baseline. ^†^ Shows a significant difference between groups. # Demonstrates a significant interaction effect. ns shows no significant interaction effect.

A significant [*F*_(3, 78)_ = 2.764, *P* = 0.047] group × time interaction effect on shear modulus was found only for RFp (Figure 3A). The shear modulus of RFp increased 29–34% at 1–3 days post-exercise (*P* ≤ 0.001) for the no-recovery group (8.6–8.9 kPa), which was significantly greater than that of the recovery group (6.7–7.1 kPa, *d* = 0.97–1.20). The shear modulus of RFd increased [*F*_(3, 78)_ = 15.792, *P* < 0.001] at 1–3 days post-exercise for both groups without a difference between groups (Figure 3B). No significant changes in shear modulus were observed for VM and VL (Figures 3C,D). When comparing the magnitude of changes among muscles and regions, a significant main effect of muscle was found [*F*_(1.980, 51.489)_ = 15.024, *P* < 0.001]. The peak changes in RFp (35 ± 24%) and RFd (43 ± 29%) were significantly greater (*P* ≤ 0.034) than those in VM (14 ± 13%) and VL (17 ± 20%).

#### Habitual Physical Activity

Physical activity data were obtained from 21 participants (*n* = 12 in the recovery group and *n* = 9 in the no-recovery group). No significant group × intensity interaction effect [*F*_(2, 38)_ = 1.103, *P* = 0.342] nor a main group effect [*F*_(1, 19)_ = 2.147, *P* = 0.159] was evident. The magnitudes of physical activity in the recovery group vs. no-recovery group were as follows: at light intensity, 596 ± 54 vs. 602 ± 41 min/day; at moderate intensity, 79 ± 25 vs. 94 ± 22 min/day; at vigorous intensity, 3 ± 4 vs. 6 ± 11 min/day.

### Correlations Between the Recovery Rate of Maximal Voluntary Isometric Contraction Torque and Other Variables

**FIGURE 4:**
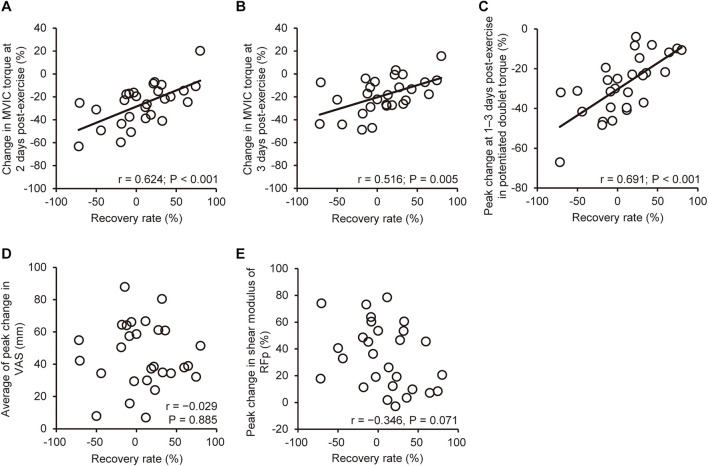
Correlations (Pearson *r*, *n* = 28) between the recovery rate of maximal voluntary isometric contraction (MVIC) torque from immediately to 1-day post-exercise and the change in MVIC torque from baseline to 2 **(A)** and 3 days post-exercise **(B)**, peak changes at 1–3 days post-exercise in potentiated doublet torque **(C)**, average muscle soreness **(D)** assessed by a visual analog scale (VAS, a 100-mm line), and shear modulus **(E)** of the rectus femoris in the proximal region (RFp).

Figure 4 shows correlations between the recovery rate of MVIC torque from immediately to 1-day post-exercise and other variables for all participants. The recovery rate of MVIC was positively correlated (*P* ≤ 0.005) with the changes in MVIC torque at 2 (*r* = 0.624) and 3 days post-exercise (*r* = 0.516), and peak change at 1–3 days post-exercise in potentiated doublet torque (*r* = 0.691). However, no significant correlations were found between the recovery rate of MVIC and the peak changes in voluntary activation, shear modulus, or muscle soreness.

## Discussion

The present study investigated characteristics of individuals who showed a recovery vs. no-recovery of MVIC torque from immediately to 1 day after a knee extensor eccentric exercise for changes in several indirect markers of muscle damage. The hypotheses were that (1) when compared to the recovery group, the no-recovery group would demonstrate greater changes in indirect muscle damage markers, and (2) the recovery rate of MVIC torque from immediately to 1-day post-exercise would be significantly correlated with the magnitude of changes in the other indirect markers of muscle damage. The results showed that the decreases in MVIC torque and potentiated doublet torque, and the increase in RFp shear modulus at 1–3 days post-exercise were greater for the no-recovery than recovery group, whereas changes in VA, muscle soreness, and shear modulus of other muscles were not different between the groups. Physical activity is a possible factor that affects the magnitude of muscle damage (Chen et al., 2011), but this effect can be excluded from the reason for the greater damage in the no-recovery than in the recovery group because of no group difference in physical activity. The recovery rate of MVIC torque was positively correlated with changes in MVIC torque from baseline to 2–3 days post-exercise and peak change in the potentiated doublet torque at 1–3 days post-exercise, but not with muscle soreness or shear modulus. These results suggest that the MVIC torque recovery rate predicts the magnitude of changes in strength variables after eccentric exercise, but does not predict other symptoms of muscle damage (muscles soreness and stiffness). Thus, the hypotheses were only partially supported.

Regarding the magnitude of changes in MVIC torque immediately after 100 maximal knee extensor eccentric contractions, the average decrease of all participants was 32% (Figure 1A). Previous studies reported an average decrease of 18% (Paschalis et al., 2011) or 42% (Doguet et al., 2016) for the MVIC torque immediately after 75 or 137 maximal eccentric contractions of the knee extensors. Crameri et al. (2007) measured MVIC torque not immediately after but 15 min after 210 eccentric contractions of the knee extensors, and reported a 16% decrease in MVIC torque. Thus, the magnitude of the MVIC torque decrease found in the present study was in the range of possible MVIC torque changes after a knee extensor eccentric exercise performed by young adults. The largest decrease in MVIC torque was reported to be at 1-day post-exercise (Crameri et al., 2007), immediately and 1-day post-exercise (Doguet et al., 2016), and at 2 days post-exercise (Paschalis et al., 2011). These were also observed in the present study when looking at individuals (Table 1). Thus, it is interesting that the recovery of MVIC torque after eccentric exercise of the knee extensors is not necessarily similar to that after eccentric exercise of the elbow flexors in which some recovery was observed from immediately to 1-day post-exercise as reported by Chen et al. (2020) and other studies (Nosaka and Sakamoto, 2001; Kim et al., 2020).

Despite the similar decline in MVIC torque immediately post-exercise between the recovery (−32.8%) and no-recovery group (−32.1%), the recovery rate of MVIC torque from immediately to 1-day post-exercise was substantially greater for the recovery group (36.5 ± 22.9%) than the no-recovery group (−25.2 ± 25.2%). It should be noted that the recovery rate showed larger variability among the participants (−71.9 to 79.9%) than the magnitude of decrease in MVIC torque from baseline to immediately post-exercise (−53.3 to −14.5%) as shown in Table 1. These suggest that the magnitude of MVIC torque decrease immediately after eccentric exercise did not necessarily indicate the magnitude of muscle damage. This was probably due to the effects of neuromuscular fatigue on the MVIC torque decrease representing combined effects of neuromuscular fatigue and damage to contractile proteins and extracellular matrix (Souron et al., 2018). Some studies attempted to find a way to predict muscle damage from the changes in variables shown immediately post-exercise. Chen et al. (2020) reported that the recovery rate of MVIC torque from immediately to 1-day post-exercise predicted the extent of muscle damage of the elbow flexors. Unlike the study by Chen et al. (2020), many participants in the current study did not show a recovery of MVIC torque from immediately to 1-day post-exercise (Table 1). This is consistent with previous studies that reported no-recovery or further decrement of MVIC torque from immediately to 1–2 days after maximal voluntary eccentric exercise of the knee extensors (McHugh and Pasiakos, 2004; Paschalis et al., 2011). However, the recovery rate of MVIC torque was positively correlated (*r* = 0.58–0.69) with the change in MVIC torque from baseline to 2–3 days post-exercise, and peak change at 1–3 days post-exercise in potentiated doublet torque from the baseline (Figure 3), supporting the findings of Chen et al. (2020). Accordingly, it seems likely that the recovery rate of MVIC torque from immediately to 1-day post-exercise is a valid predictor for changes in neuromuscular indices following eccentric exercise, irrespective of muscle groups.

At 1–3 days post-exercise, MVIC torque and potentiated doublet torque were lower for the no-recovery than recovery group, whereas VA was not different between the groups (Figure 1). The time-course of changes in MVIC torque has been shown to be more associated with peripheral than central factors (Behrens et al., 2012; Doguet et al., 2016). This was also found in the present study showing that significant changes at a time from baseline were consistent between MVIC torque and potentiated doublet torque, whereas a significant decrease in VA was limited to immediately post-exercise. These results suggest that peripheral rather than central neuromuscular function affected the MVIC torque reduction after 1 day post-exercise.

Previous studies reported an increase in muscle shear modulus after eccentric exercise (Lacourpaille et al., 2014; Ema et al., 2021) and suggested that an increase in muscle shear modulus represents muscle damage (Lacourpaille et al., 2017). Thus, we expected greater increases in muscle shear modulus in the no-recovery group and fast a recovery for the recovery group. In the current study, changes in RFp shear modulus were greater for the no-recovery than the recovery group (Figure 3). Guilhem et al. (2016) reported that the individual variability of maximal fascicle length changes during eccentric exercise was correlated with the variability of MVIC torque decrease at 2 days post-exercise. Thus, it is possible that a difference in fascicle behavior existed between the recovery and no-recovery groups. Further studies are needed to clarify this point. It is likely that the greater decrease in MVIC torque in the no-recovery than recovery group was due to greater damage to RF. It should be noted that despite the significant group difference in the changes in RFp shear modulus, peak change in the modulus was not associated with the recovery rate of MVIC torque (Figure 3). The RF muscle volume is the smallest among the constituents of the quadriceps femoris, with its proportional contribution to the total quadriceps femoris being not more than approximately 15% (Ema et al., 2018; Akagi et al., 2020). Therefore, a possible impact of individual variability of RF damage on the variability of MVIC torque may have been masked, resulting in insignificant correlations between the recovery rate and change in RFp shear modulus.

The recovery rate of MVIC torque was not associated with the changes in muscle soreness (Figure 3), which contradicted by the findings of the study by Chen et al. (2020) who reported a significant correlation between the recovery rate of MVIC torque and muscle soreness (*r* = −0.611). However, Chen et al. (2020) investigated responses of indirect muscle damage markers induced by three different eccentric exercises (>1,000 contractions at 10%MVIC;>20 contractions at 50%MVIC;>12 contractions at 100%MVIC). Muscle soreness was greater after high- than low-intensity eccentric exercise with matched mechanical work (Mavropalias et al., 2020). DOMS is considered to be more related to damage and inflammation to non-contractile than contractile tissues (Crameri et al., 2007), suggesting that underpinning mechanisms of the exercise-induced changes are different between MVIC torque and muscle soreness. Therefore, it is possible that the associations between the recovery rate of MVIC torque and muscle soreness found by Chen et al. (2020) reflected task-dependent characteristics of muscle damage. We investigated the adaptability of the recovery rate of MVIC torque as a predictor of muscle damage when performing the same eccentric exercise among individuals with large inter-individual variability in recovery rate. The recovery rate could predict MVIC and evoked torque following the eccentric exercise, but that could not predict the extent of changes in muscle shear modulus (the index of muscle stiffness) and muscle soreness. Thus, a limitation exists for the use of the recovery rate of MVIC as a predictor of muscle damage symptoms.

There were several limitations in the present study. Firstly, muscle damage was evaluated using indirect markers only. Thus, possible group differences in and association with the recovery rate and the histological changes in muscle fibers remain unclear. Secondly, only MVIC torque at a supine position was evaluated in the current study. Paschalis et al. (2011) reported greater decreases in eccentric contraction torque than isometric and concentric contraction toque in a sitting position at immediately (−21% vs. −18%,−10%) and 2 days (−31% vs. −24%,−22%) after 75 maximal eccentric contractions of the knee extensors. At 1–3 days post-exercise after 100 maximal eccentric contractions of the knee extensors, the magnitude of MVIC torque decrease was greater in a sitting than a supine position (Ema et al., 2021). Therefore, a more clear prediction of muscle damage might be possible if the recovery rate of dynamic contraction torque and/or MVIC torque is measured at a sitting position. Thirdly, we determined indirect markers of muscle damage only up to 3 days post-exercise. Consequently, there were many variables that did not recover to their baseline values in 3 days post-exercise, and we were not able to follow these variables until they were fully recovered. However, MVIC torque recovered to its baseline value by 3 days post-exercise for the recovery group. Hence, the lack of measurement data after 4 days post-exercise will not have a significant impact on the interpretation of the current results. Lastly, the exact causes of the differences in the changes of the dependent variables between the recovery and no-recovery groups were not clear from the present study. A large variability of responses to eccentric exercise is documented (Doguet et al., 2016), and previous exposures to eccentric contractions (Ema et al., 2021), genetic factors (Kim et al., 2020), and other factors such as gender, age, and nutrition (Tsuchiya et al., 2021) affect the responses. It is interesting to investigate further the mechanisms underpinning the individual differences in the MVIC torque recovery and other symptoms of muscle damage.

## Conclusion

The present study found large individual differences in the recovery rate of MVIC torque from immediately to 1 day after knee extensor eccentric exercise. Compared with the participants who showed recovery of the MVIC torque from immediately to 1-day post-exercise (the recovery group; *n* = 15), decreases in MVIC torque and potentiated doublet torque, and increase in RFp shear modulus were greater for the participants who did not show the MVIC torque recovery from immediately to 1-day post-exercise (the no-recovery group; *n* = 13) with a significant decrease in voluntary activation only at immediately after the exercise. Changes in voluntary activation, muscle soreness of the investigated muscles, and shear modulus of RFd, VM, and VL were not different between the groups. It appears that peripheral rather than central neuromuscular function affected the MVIC torque reduction. The recovery rate of MVIC torque from immediately to 1-day post-exercise was positively correlated with the change in MVIC torque from baseline to 2–3 days post-exercise, and peak change in potentiated doublet torque at 1–3 days post-exercise from the baseline, but was not correlated with changes in the other dependent variables. Thus, the MVIC torque recovery rate can be used for the prediction of exercise-induced changes in maximal voluntary strength and evoked strength, but cannot predict the extent of DOMS and muscle stiffness changes after eccentric exercise of the knee extensors.

## Data Availability Statement

The raw data supporting the conclusions of this article will be made available by the authors, without undue reservation.

## Ethics Statement

The studies involving human participants were reviewed and approved by the Ethics Committee of Shibaura Institute of Technology. The patients/participants provided their written informed consent to participate in this study.

## Author Contributions

MS, RE, KN, KH, and RA conceived and designed the experiments. MS, RE, AK, KH, and RA performed the experiment. MS, RE, KH, and RA analyzed the data. MS and RE drafted the manuscript and prepared tables and figures. All authors interpreted the results of the research, edited, critically revised, and approved the final version of the manuscript, and have agreed to be accountable for all aspects of the work related to its accuracy and integrity.

## Conflict of Interest

AK was employed by Mizuno Corporation. The remaining authors declare that the research was conducted in the absence of any commercial or financial relationships that could be construed as a potential conflict of interest.

## Publisher’s Note

All claims expressed in this article are solely those of the authors and do not necessarily represent those of their affiliated organizations, or those of the publisher, the editors and the reviewers. Any product that may be evaluated in this article, or claim that may be made by its manufacturer, is not guaranteed or endorsed by the publisher.
